# Effects of intensive physiotherapy on Quality of Life (QoL) after pancreatic cancer resection: a randomized controlled trial

**DOI:** 10.1186/s12885-022-09586-1

**Published:** 2022-05-09

**Authors:** Dirk Weyhe, Dennis Obonyo, Verena Uslar, Navid Tabriz

**Affiliations:** Carl von Ossietzky University Oldenburg, University Hospital for Visceral Surgery, Pius-Hospital Oldenburg, Georgstr. 12, 26121 Oldenburg, Germany

**Keywords:** QLQ-C30, QLQ-PAN26, Early mobilization, Home-based exercise, Long-term follow-up

## Abstract

**Background:**

Patients have significantly lower QoL scores after pancreatic resection due to cancer in the physical and psychological domains compared to healthy controls or other cancer patients. Intensified physiotherapy or physical training can increase QoL by reducing fatigue levels and improving physical functioning. However, data on the long-term effects of intensive or supervised physiotherapy is lacking. The aim of this exploratory study is the assessment of QoL in the intervention group, using various QoL questionnaires in their validated German translations and gather data on its feasibility in the context of chemotherapy with a follow-up of 12 months (and develop concepts to improve QoL after pancreatic cancer resection).

**Methods:**

Fifty-six patients (mean age: 66.4 ± 9.9 years) were randomized in this study to intervention (cohort A, *n* = 28) or control group (cohort B, *n* = 28). Intervention of intensified physiotherapy program consisted of endurance and muscle force exercises using cycle ergometer. In the control group physiotherapy was limited to the duration of the hospital stay and was scheduled for 20 min on 5 days per week. The clinical visits took place 2 days preoperatively, 1 week, 3 months, 6 months and 12 months postoperatively. Both groups attended the follow-up program. QoL was evaluated using the Short Physical Performance Battery (SPPB), Short Form-8 Health Survey (SF-8) and the European Organization for Research and Treatment of Cancer (EORTC) QLQ-C30 and pancreatic cancer‐specific module QLQ-PAN26 questionnaires. The course of QoL was evaluated using a repeated measures ANOVA and a per protocol design.

**Results:**

Of the initial 56 randomized patients, 34 finished the 12 months follow-up period. There were no adverse events due to the intervention and 80% of patients in the intervention group where adherent. There was no significant influence on physical performance as measured by SPPB and SF-8 questionnaire. However, after 6 months patients in the intervention group regained their prior physical condition, whereas the control group did not. Intensive physiotherapy significantly influenced various factors of QoL measured with the C30 questionnaire positively, such as physical functioning (*p* = 0.018), role functioning (*p* = 0.036), and appetite loss (*p* = 0.037), even after 6 months. No negative effects in patients undergoing chemotherapy compared to those without chemotherapy was observed.

**Conclusion:**

This first randomized controlled study with a 12-month follow-up shows that supervised physiotherapy or prescribed home-based exercise after pancreatic cancer resection is safe and feasible and should be proposed and started as soon as possible to improve certain aspects of QoL.

**Trial Registration:**

German Clinical Trials Register (No: DRKS00006786); Date of registration: 01/10/2014.

## Background

Pancreatic cancer is the 12th most common cancer worldwide and the fourth leading cause of cancer-related death, behind lung, colorectal and breast cancer in Europe 2018 [1, 2]. Currently surgery combined with chemotherapy offers the only chance for cure, though with a 5-year survival ranging from 8–17% [3]. This seems mostly due to the fact that 80%-85% of the patients are diagnosed at locally advanced or metastatic stage [4, 5]. Especially in the light of this short overall survival, it seems important to ensure good QoL for the remainder of the patients life. However, surgery itself, and the following adjuvant therapy seem to have a large impact on QoL [6]. Also, 65–97% of the patients with pancreatic cancer already present with frailty or fatigue, sarcopenia and / or weight loss, which generally reduces patients QoL even prior to surgery [7, 8], and it is also known that these factors have been associated with poor overall survival [9, 10].

The clinical and scientific interest in the quality of life (QoL) of patients with cancer has been increasing since the 1990s [11]. It has been shown that intensified physical activity can measurably increase QoL, i.e. by improving or restoring muscle strength, physical functioning and reduce fatigue levels of patients with cancer [7, 12–14]. A few studies that have dealt with the issue of QoL and developed concepts to improve the QoL of patients after pancreatic resection [7, 15–17]. Yeo et al. found that patients with pancreatic and periampullary cancer who have undergone resection benefit from a structured walking program with respect to fatigue, pain, physical functioning, and mental health [7]. Steindorf and colleagues assessed the efficacy of 6-month resistance training on physical functioning and QoL-related outcomes in 65 pancreatic cancer patients [17], of which 47 patients completed the 6-month intervention phase After 6 months no effects of resistance training were observed. Nonetheless, data for QoL after 6 months is still missing, even though chemotherapy is known to influence QoL, and patients often receive it for a longer period of time. Chemotherapy influences several domains of QoL either negatively (i.e. nausea, loss of appetite and global health status)[18] or positively as some subscales of QoL improve during the course of chemotherapy, i.e. physical and emotional functioning, pain and sleeping disturbances [19].

Based on the already published trial protocol, we are to our knowledge one of the first to investigate the effect of intensive physiotherapy after pancreatic cancer resection with a follow-up of 12 months [20]. The primary aim of this study is to investigate whether intensive physiotherapy, consisting of a combination of endurance and muscle force exercises, improves the QoL of patients after resection of pancreatic cancer or tumors, using disease-specific and non-specific questionnaires over the course of the follow-up in comparison to standard physiotherapy or usual care. The secondary aims of this study included analysis of the subscales of the questionnaires to determine which aspects of QoL suffer most after surgery, and physical performance. Further, influence on 1-year survival rate, study drop outs, and tumour recurrence rate after 6 and 12 months were documented. The overall goal was to gather data which should enable the development of concepts to improve the QoL after pancreatic cancer resection, and evaluate in an exploratory way which questionnaires are most sensitive for this kind of studies in the future.

## Methods

The study was approved by the medical ethics committee of the Carl von Ossietzky University Oldenburg (No: 59/2014). It was conducted in accordance with the Declaration of Helsinki. The study was registered with the German Clinical Trials Register (No: DRKS00006786; Date of registration: 01/10/2014) and conformed to the CONSORT (Consolidated Statement of Reporting Trials) 2010 statement [21]. For further details we also refer to our publication of the study protocol [20].

### Study population

This study was conducted as a prospective randomized interventional study and consisted of patients scheduled for surgery for resectable pancreatic cancer or tumours. Further inclusion criteria were age of ≥ 18 years and surgery and initial postoperative hospitalization in the Clinic of General and Visceral Surgery, Pius-Hospital Oldenburg, Germany. Recruitment started on 01.02.2016 and ended on 20.11.2018. The follow-up period ended on 28.11.2019. Recruitment stopped as planned, when the number of participants needed was reached.

Exclusion criteria were lack of written consent, physical inability to participate in the intensified physiotherapy program, illiteracy, inability of the German language, physical or mental disability that precludes participation in the intensified physiotherapy program, and lack of compliance (for adherence criteria see below). Individual criteria for discontinuation were withdrawal of the patient’s consent to study participation, new cardiac risk profile as myocardial infarction or congestive heart failure, complicated course after pancreatectomy as postoperative mechanical ventilation over the first postoperative week, and physical or mental incapacity to participate in the intensified physiotherapy program. All enrolled patients provided a written informed consent.

### Study design

Included patients were randomized in an interventional group (intensified physiotherapy = cohort A) and control group (standard physiotherapy = cohort B). For this purpose, a MATLAB-based script was used that generated a list for 84 study enrolments with random permuted blocks and a block size of 6 with 1:1 allocation. Based on this list, 84 numbered envelopes were prepared by a person not directly involved in this study, each containing a slip of paper with the group assignment. These opaque envelopes were opened in consecutive order in the presence of the patients after inclusion.

**Fig. 1:**
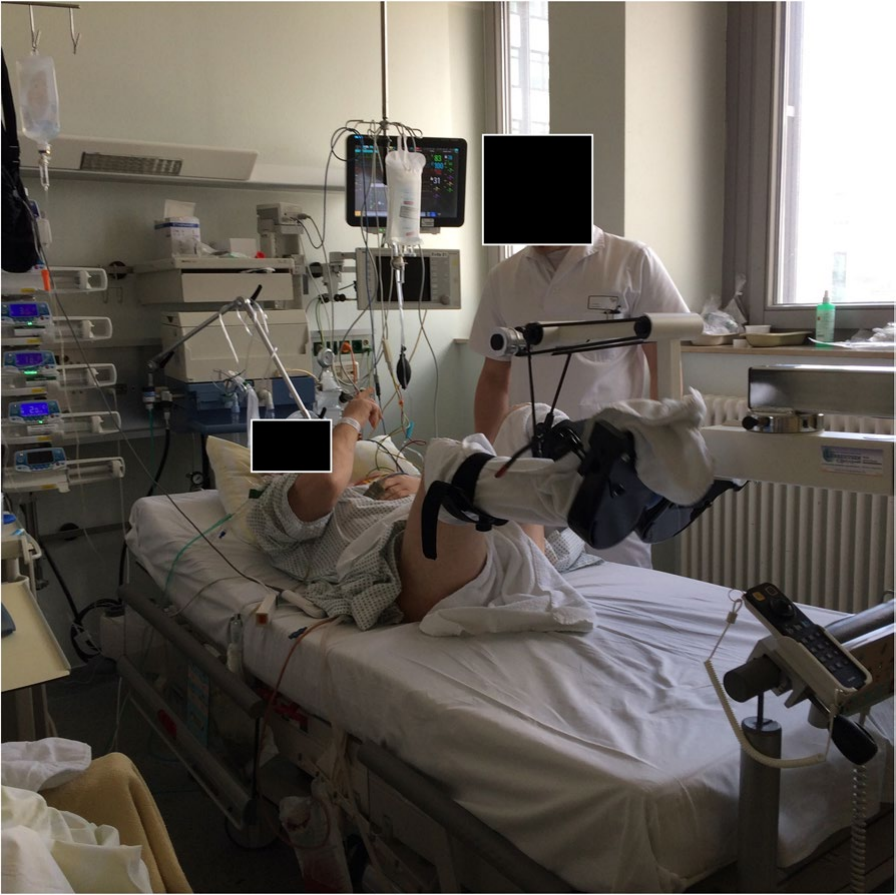
Patient with the bed bicycle

The intensified physiotherapy of cohort A took place as follows: It started within the first 24 h after extubating with 3 rounds of in-bed cycling per day, each round taking 10 min (see also Fig. 1). From the second postoperative week, patients were requested to walk 3 times/day (15 min each), perform muscle exercises using a “Theraband” (resistance band) and cycle ergometer, 2-kg dumbbells and modified squats 5 days per week as described by our study protocol [21]. Physiotherapists were instructed to respect the study design and were briefed monthly. The experienced physiotherapists used their clinical judgement to observe the feasibility and practicability of the exercise to ensure patient safety. Adherence of the physiotherapists conducting the intensified training was checked by use of a list the physiotherapist had to fill out during and after each training session, describing the duration and intensity of the training. One of the authors talked personally or on telephone once a week with the physiotherapists to inquire about any difficulties and check on adherence in order to avoid deviations from the study design.

After discharge from the rehabilitation clinic to the 12th postoperative month, cohort A patients were encouraged to walk according to the Every Step Counts graduated walking program with Yeo et al.’s minor modifications for resected pancreas and periampullary cancer patients [7], and they are asked to continue their muscle exercises at least 3 times per week (i.e. 15–20 min for each session). To encourage walking and other physical exercises, patients in cohort A received a pedometer (HiTrax Walk by TFA Dostmann GmbH & Co. KG), a Theraband and a diary where they documented the daily steps and other physical activities. The standard physiotherapy in cohort B depended on the physical condition and willingness of the patient and was limited to the duration of the hospital stay and was scheduled for 20 min on 5 days per week. It consisted of individual therapy with relaxation and mobilization exercises, walking, and possibly even climbing stairs. At discharge, patients in cohort B also received the diary but no pedometer or Theraband. Both groups were subjected to a 12 month follow-up program. To minimize rehabilitation therapy as a confounding factor, the majority of our patients received rehabilitation in the same clinic. Patients were called once monthly by study nurses via telephone and asked the following questions: 1. Do you perform the prescribed intensified physiotherapy? 2. If not, for what reason? Further, current medical condition or on-going therapy, including possible treatment-related side effects, were recorded. Patients not adhering to the program were excluded from the analysis. Adherence was defined as attendance and completion of scheduled or prescribed sessions. However, all patients were followed-up until death or until the end of the follow-up period. The rate or extent of adherence was checked after the last follow-up by screening the diaries of the patients and the physiotherapists upon their return.

### Study outcomes

The primary outcome was the QoL after 12 months, as measured by the SF-8, and the disease-specific EORTC QLQ-C30 / QLQ-PAN26 questionnaires in the validated German translation in each cohort [11, 22–24].

The secondary end points were study dropouts, and physical performance measured by the "Short Physical Performance Battery" (SPPB) [25]. Furthermore, influence on 1-year survival rate, effects of potential adjuvant therapy on QoL, and tumour recurrence rate after 6 and 12 months were as well investigated. The longitudinal development of QoL was measured by the SF-8 and the QLQ-C30/ PAN26 questionnaires. The different domains of the mentioned questionnaires were evaluated separately.

The clinical visits took place 2 days preoperatively (day of inclusion), 1 week, 3 months, 6 months and 12 months postoperatively. At the first visit patients were asked about their typically physical activities and intensity before their diagnosis. All visits included the following measures: assessment of QoL with the three questionnaires, documentation of medication and adjuvant treatment as well as documentation of the tumour markers CEA and CA 19–9, assessment of mobility using the SPPB [25], and nutritional status. Further, the following parameters were measured: body mass index (BMI) and body fat measurement by using the seven-site skinfold method. Results of those parameters and of the nutritional status are reported in depth elsewhere [26]. Additionally, CT scans of the abdomen were performed at 6 and 12 months postoperatively.

### Statistical analysis

Data was analysed descriptively by calculating means and standard deviations, or numbers and percentages. For patient characteristics, for nominal and ordinal scaled data either Chi^2^ or Fishers exact test were used (depending on the number of patients in any one cell of the respective contingency tables), and for interval scaled data the Mann–Whitney U test was used since the Shapiro–Wilk test was significant for all variables, indicating non-normally distributed data. For survival, mean overall survival and respective Confidence Intervals (CI) were calculated. Data for the outcomes SPPB, and SF-8, C30 and PAN26 subscales were analysed using IBM SPSS 26 with per protocol analyses in a repeated measures ANOVA design. In case of significant *p*-values (*p* ≤ 0.05) for differences between cohorts’ post-hoc tests (tukey test) were conducted to evaluate for which visits there was a significant difference between cohorts. Benjamini–Hochberg correction was applied for multiple testing using an online tool [27, 28]. All *p*-values given below are the corrected values. Since this was an exploratory study, only post-hoc power calculations, based on the data gathered here, were conducted to establish number of cases needed for future studies with GPower Version 3.1.

## Results

### Patient characteristics

**Fig. 2:**
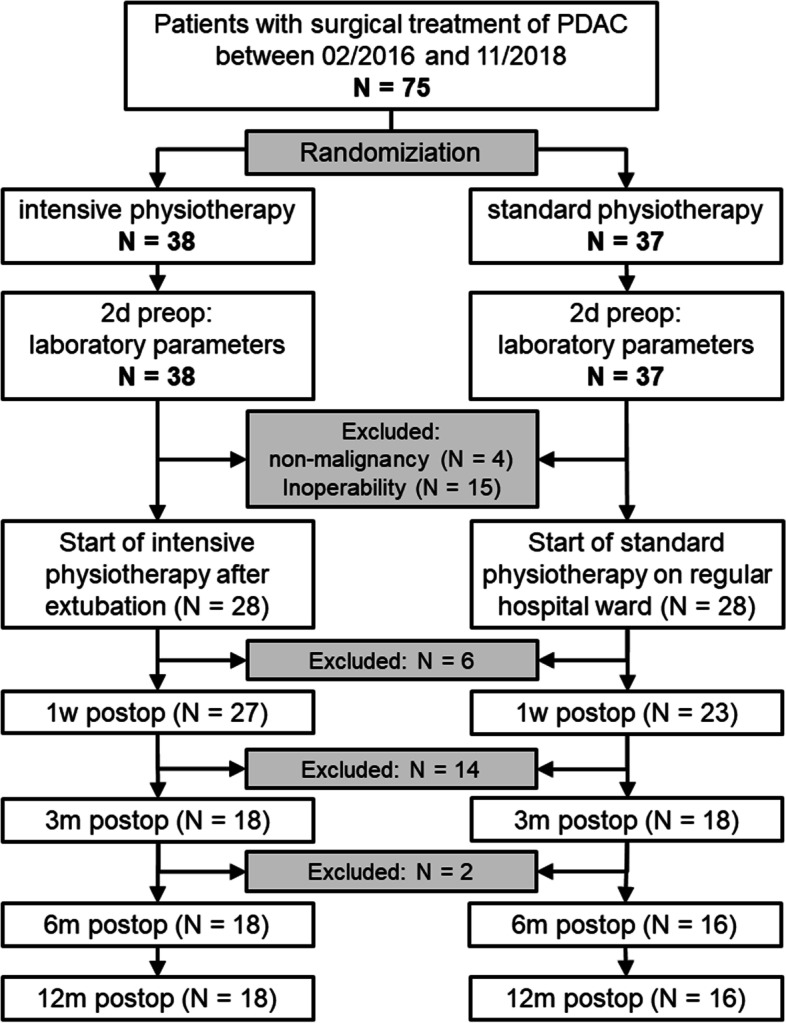
Study Flow Chart; *One patient of cohort B dropped out at their own request before the 1-week postoperative visit. Three patients of cohort B and one of cohort A dropped out at their own request at some point between the 1-week and the 3-month postoperative visit. All other drop outs (*n* = 17) were due to the death of the patient

Seventy-five patients were considered to have resectable tumours and invited to participate in this study. Of these, 38 patients were randomized in the intervention group (cohort A), and 37 were randomized into the control group (cohort B; see also study flow chart in Fig. 2). The cohorts were comparable with regards to their prior workout routine and physical activities. Nineteen patients were excluded postoperatively (10 in cohort A; 9 in cohort B) because either the histopathology showed non-malignant results, or patients were unresectable. Patient characteristics of the remaining 56 patients are displayed in Table 1. There were no relevant differences between both groups, which might serve as confounders with regards to the main outcomes. Reoperations were needed in 9 cases (equivalent to Calvien Dindo grade IIIb-IV), with 7 of them because of anastomotic leakage, and 2 being explorations because of elevated markers of inflammation, but no complications were detected during exploration. Five minor complications (Clavien Dindo grade ≤ II) were recorded, i.e., haematoma (*n* = 2) or biochemical leakage (*n* = 3) which were treated conservatively. Thirty-four patients completed the 12-month follow-up period (18 in cohort A, 16 in cohort B). The drop-out rate was 36% (10 patients) in cohort A and 43% (12 patients) in cohort B. Most drop-outs (*n* = 10 in cohort A, and *n* = 7 in cohort B) were due to the death of the patients (see also Table 1). Five patients dropped out at their own request (1 in cohort A; 4 in cohort B). In cohort A 36% of patients died during the course of the follow-up, and 25% in cohort B. No adverse events relating to the prescribed exercise was observed. Mean overall survival was 23.2 months (95% CI: 16.1 – 30.2 months) for cohort A, and 24.2 months (17.5 – 30.9 months) for cohort B.

**Table 1:** Patient characteristics

	overall	cohort A	cohort B	*p*-value*
	*n* = 56	*n* = 28	*n* = 28	
sex (m/f)	33/23	18/10	15/13	0.666
age (years; mean ± SD)	66.4 ± 9.9	68.0 ± 8.9	64.8 ± 10.8	0.666
BMI (mean ± SD)	26.8 ± 5.2	25.9 ± 4.7	27.0 ± 5.5	0.705
ASA (n)				0.666
1	1	1	0	
2	18	8	10	
3	34	19	15	
4	1		1	
type of surgery (n)				0.666
pylorus preserving	47	22	25	
pancreatic left resection	9	6	3	
carcinoma type (n)				0.666
Adenocarcinoma	48	23	25	
NET	4	3	1	
IPMN carcinoma	3	3	0	
Acinar cell carcinoma	1		1	
UICC stadium (n)				0.666
IA	2	1	1	
IB	6	3	3	
IIA	10	8	2	
IIB	22	7	15	
III	10	4	6	
IV	4	4	0	
CRM status (R0 > 1 mm/R1 ≤ 1 mm)	32/24	18/10	14/14	0.666
CTx received (n)	39	18	21	0.666
complications				0.666
Clavien Dindo Grade III-IV	7	2	5	
Clavien Dindo < III	5	1	4	
ICU stay (days; mean ± SD)	6.3 ± 5.5	5.4 ± 2.9	7.2 ± 7.3	0.666
Hospital stay (days; mean ± SD)	23.0 ± 17.8	20.0 ± 8.0	26.4 ± 24.3	0.666
recurrence (n)	27	15	12	> 0.999
30 day mortality (n)	5	2	3	> 0.999
death during follow-up (n)	17	10	7	0.666

*NET* Neuroendocrine Tumour

*IPMN* Intraductal Papillary Mucinous Neoplasm-Associated Carcinoma

*CRM* circumferential resection margin

*CTx* Chemotherapy

*BMI* Body Mass Index

*ASA* American Society of Anesthesiologists Physical Status classification

*UICC* Union for International Cancer Control

*ICU* Intensive Care Unit

^*^corrected *p*-values (false discovery rate); for ordinal- or nominal-scaled data the Chi^2^-test or the Fisher exact test were used, for interval-scaled data, Mann–Whitney U test was used

With regards to adherence to the prescribed protocol during hospital stay, mean postoperative hospital stay of 20 days, resulting in about 50 to 60 appointments (typically three times a day) with the physiotherapists and self-conducted workout sessions per patient. According to the documentation provided by the physiotherapists and the patients, about 50% of all patients in cohort A missed up to 5 sessions during the course of the hospital stay, mostly due to temporary discomfort. However, no patient missed more than 10 sessions of physiotherapy. According to the exercise diaries handed out to all patients for their home-based prescribed exercise, 80% of patients in cohort A adhered to their prescribed exercises throughout the course of the whole follow-up, and patients in cohort B often took up their preoperative exercise behaviour sometime after discharge from hospital.

### Physical assessment according to SPPB

**Fig. 3:**
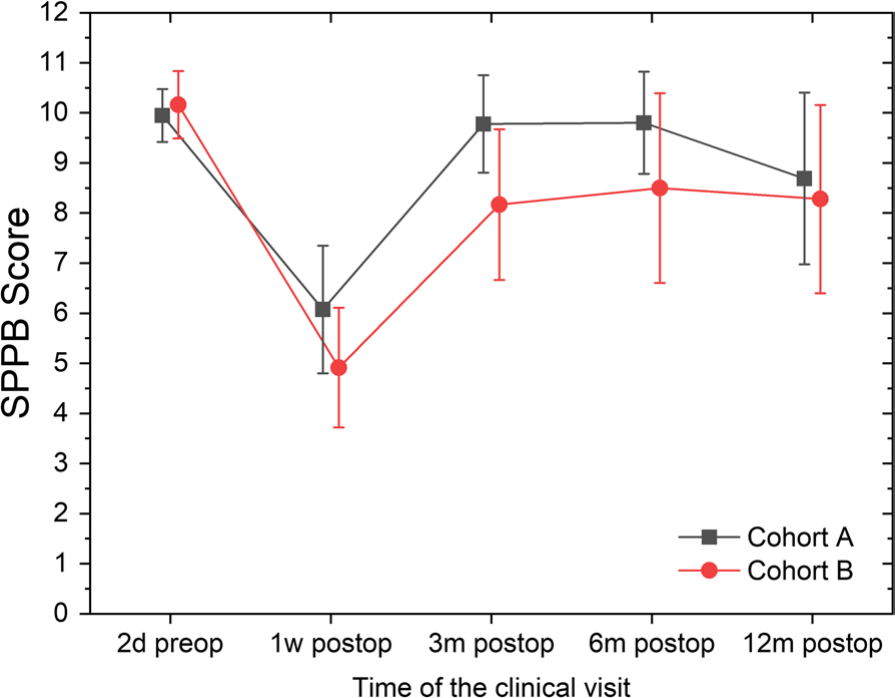
Mean and 95%CI for the mean for the Short Physical Performance Battery Score split by clinical visit and cohorts. Higher values indicate better performance

Physical assessment using the SPPB was comparable between groups at the first visit. This is consistent with the fact that self-reported physical activity before the onset of the disease and the nutritional status measured at the first visit was comparable between the groups. Overall, intensive physiotherapy has no significant influence on physical performance as measured by SPPB (F(1,26) = 0.684; *p* = 0.416; see Fig. 3). However, after 3 and 6 months patients in cohort A almost regain their physical condition comparable with before the operation, despite chemotherapy taking place during that time for most patients (*n* = 18 in cohort A, and *n* = 21 in cohort B), whereas performance in cohort B tends to stay lower.

### Quality of Life assessment according to SF-8

**Fig. 4:**
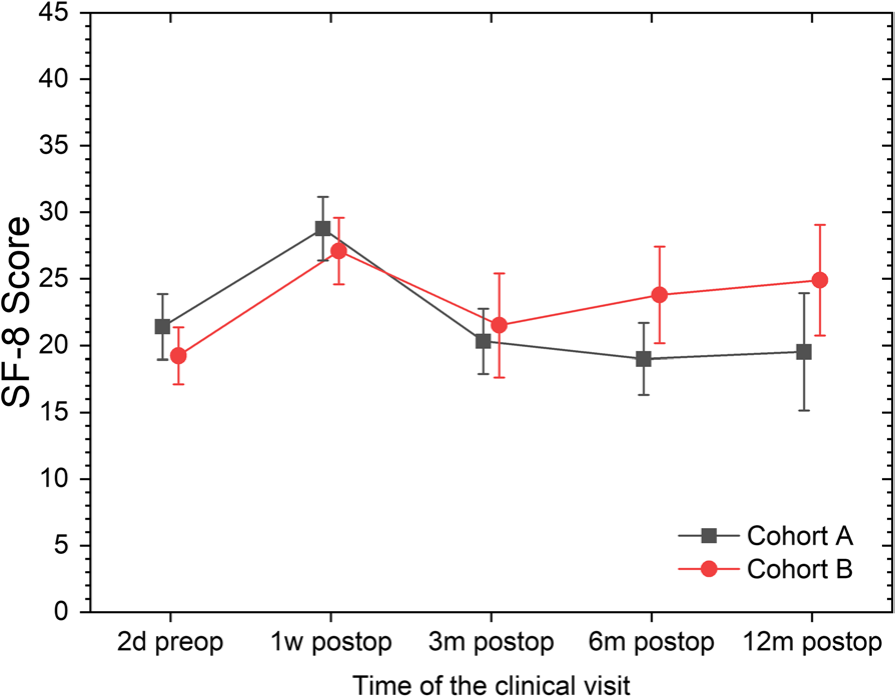
Mean and 95%CI for the mean for the SF-8 QoL Score split by clinical visit and cohorts. Lower values indicate better Qol

The assessment according to SF-8 showed no significant difference between the two cohorts (Fig. 4). However, after 6 and 12 months there was a noticeable difference between the cohorts, with cohort A reaching preoperative level or better, and cohort B not reaching preoperative QoL.

### Quality of Life assessment according to EORTC QLQ-C30

The assessment according to the QLQ-C30 showed no significant differences in the Repeated Measures ANOVAs between cohorts for the subscales Global Health Status, emotional functioning, cognitive functioning, social functioning, nausea and vomiting, pain, dyspnoea, insomnia, appetite loss, constipation, and diarrhoea (Table 2). Significant differences between cohorts could only be found for:

- appetite loss (F(1,31) = 4.761; *p* = 0.037; effect size η^2^_p_ = 0.133), with post-hoc tests only being significant 1 week postoperatively (*p* = 0.038),
- physical functioning (F(1,31) = 6.269; *p* = 0.018; η^2^_p_ = 0.168), with post-hoc tests being significant from 3—12 months postoperatively (*p* < 0.018 for all visits)
- role functioning (F(1,26) = 4.903; *p* = 0.036; η^2^_p_ = 0.159), with post-hoc tests being significant after 6 to 12 months (*p* = 0.038, and *p* = 0.014, respectively).

**Table 2:** Means and 95% CI for each QoL Questionnaire and the SPPB for each visit stratified by cohort. The *p*-value for the between subjects effect of cohorts as calculated by the Repeated Measures ANOVA is displayed as well (italics indicate significant differences) as the number of patients needed to achieve 1 - ß > 0.8 according to post-hoc power analysis

	**2d preoperative**		**1w postoperative**		**3 m postoperative**		**6 m postoperative**		**12 m postoperative**		***p*-value (RM ANOVA)**	**n needed for 1 - ß > 0.8**
	Cohort A	Cohort B	Cohort A	Cohort B	Cohort A	Cohort B	Cohort A	Cohort B	Cohort A	Cohort B		
**SPPB**	9.9 (9.4;10.5)	10.2 (9.5;10.8)	6.1 (4.8;7.3)	4.9 (3.7;6.1)	9.8 (8.8;10.7)	8.2 (6.7;9.7)	9.8 (8.8;10.8)	8.8 (6.6;10.4)	8.7 (7.0;10.4)	8.3 (6.4;10.2)	.416	~ 120
**SF-8**	22.1 (19.7;24.4)	19.0 (16.8;21.3)	28.5 (26.3;30.7)	27.5 (24.6;30.4)	19.4 (15.8;23.0)	22.7 (19.9;25.5)	20.7 (17.9;23.6)	21.7 (17.7;25.7)	23.3 (19.0;27.6)	21.0 (16.5;25.5)		
**C30 questionnaire**												
**Global Health Status**	55.5 (46.5;64.4)	62.9 (55.6;70.2)	34.9 (26.1;43.6)	40.7 (31.4;50.0	64.4 (55.1;73.6)	55.2 (44.9;65.4)	65.4 (54.9;75.9)	54.8 (43.7;65.8)	62.3 (49.8;74.7)	50.0 (37.9;62.1)	.458	~ 100
**Physical functioning**	79.0 (70.2;87.8)	81.8 (74.7;88.9)	36.3 (24.9;47.7)	25.8 (14.5;37.2)	***78.5 (69.8;87.2)***	***59.3 (47.0;71.6)***	***79.0 (69.1;88.9)***	***57.8 (45.5;70.0)***	***78.8 (68.3;89.3)***	***55.8 (41.5;70.1)***	***.018***	~ 19
**Role functioning**	68.1 (56.3;80.0)	76.0 (65.6;86.4)	22.0 (9.8;34.2)	19.7 (4.4;35.0)	67.4 (54.3;80.6)	54.0 (37.1;70.8)	***70.8 (56.2;85.4)***	***46.8 (30.6;63.0)***	***73.5 (58.2;88.9)***	***43.9 (27.8;59.9)***	***.036***	~ 36
**Emotional functioning**	57.8 (47.7;67.9)	58.8 (48.5;69.1)	57.7 (46.2;69.3)	48.2 (35.7;60.7)	70.5 (58.8;82.1)	50.4 (34.4;66.4)	70.4 (58.1;82.8)	58.3 (46.1;70.6)	67.2 (54.4;79.9)	53.5 (37.0;70.0)	.287	~ 100
**Cognitive functioning**	80.2 (70.7;89.8)	76.0 (67.7;84.3)	64.8 (53.2;76.4)	58.7 (46.1;71.2)	81.8 (71.8;91.9)	71.4 (58.6;84.2)	82.5 (73.2;91.8)	67.5 (56.5;78.4)	73.5 (59.2;87.8)	64.9 (52.2;77.6)	.350	~ 100
**Social functioning**	68.2 (55.7;80.8)	76.0 (65.9;86.0)	44.9 (30.4;59.3)	43.1 (25.9;60.2)	67.4 (53.6;81.3)	69.0 (53.5;84.5)	70.0 (57.6;82.5)	60.3 (43.5;77.1)	71.6 (56.8;86.3)	53.5 (36.8;70.2)	.653	~ 100
**Fatigue**	40.0 (30.3;49.7)	37.9 (27.5;48.3)	72.0 (60.7;83.3)	68.9 (57.5;80.3)	38.4 (28.4;48.4)	53.4 (39.0;67.9)	40.0 (28.5;51.5)	62.4 (49.9;75.0)	37.9 (27.7;48.1)	55.0 (40.6;69.3)	.256	~ 100
**Nausea and vomiting**	15.2 (7.6;22.8)	10.8 (4.6;16.9)	28.4 (16.1;40.7)	16.7 (4.4;28.9)	8.3 (1.3;15.4)	13.5 (1.3;15.4)	9.2 (-0.7;19.1)	11.1 (0.2;22.0)	7.8 (-4.1;19.8)	5.3 (0.9;9.6)	.563	> 600
**Pain**	27.5 (16.7;38.2)	28.9 (17.2;40.6)	56.2 (43.2;69.1)	37.3 (26.1;48.5)	22.7 (10.8;34.6)	33.3 (18.2;48.5)	30.0 (17.8;42.2)	35.7 (21.2;50.2)	24.5 (9.7;39.4)	38.6;26.1;51.1)	.529	~ 100
**Dyspnoea**	30.3 (18.1;42.5)	22.6 (11.1;33.5)	48.2 (35.4;60.9)	42.7 (28.8;56.5)	33.3 (19.1;47.6)	28.3 (13.9;42.8)	33.3 (19.9;46.7)	36.5 (22.3;50.7)	21.7 (5.8;37.4)	36.8 (20.4;53.3)	.906	> 1400
**Insomnia**	47.1 (33.8;60.3)	36.3 (24.5;48.1)	66.7 (53.6;79.7)	53.3 (37.8;68.9)	37.9 (21.1;54.7)	50.8 (36.8;64.8)	36.7 (21.8;51.6)	50.0 (33.9;66.1)	39.2 (24.1;54.3)	59.7 (41.2;78.1)	.548	~ 34
**Appetite loss**	35.3 (23.6;47.0)	21.2 (11.0;31.4)	***60.5 (48.4;72.6)***	***40.0 (25.4;54.6)***	25.8 (12.2;39.3)	22.2 (7.0;37.4)	23.3 (9.0;37.6)	19.0 (3.7;34.4)	13.7 (1.1;26.3)	18.5 (7.7;29.4)	***.037***	> 750
**Constipation**	19.6 (8.9;30.3)	17.7 (6.9;28.4)	41.3 (24.8;57.9)	33.3 (17.3;49.3)	21.2 (7.3;35.2)	15.9 (5.2;26.6)	11.7 (1.9;21.5)	11.1 (2.9;19.3)	11.8 (4.0;19.6)	17.5 (3.1;32.0)	.248	~ 100
**Diarrhoea**	26.0 (15.2;36.9)	32.4 (20.5;44.2)	17.3 (6.6;28.0)	24.0 (8.7;39.3)	10.6 (2.7;18.5)	27.0 (11.6;42.4)	18.3 (7.2;29.4)	34.9 (19.6;50.2)	17.7 (2.7;32.6)	24.6 (7.4;41.8)	.106	~ 100
**Financial difficulties**	7.3 (0.3;14.3)	9.4 (2.1;16.7)	11.1 (2.6;19.7)	12.5 (2.2;22.8)	22.7 (7.0;38.5)	16.7 (7.8;25.5)	23.3 (9.0;37.6)	28.1 (12.1;44.1)	9.8 (0.5;19.1)	28.1 (12.9;43.3)	.319	> 750
**PAN26 questionnaire**												
**Pancreatic Pain**	25.5 (17.4;33.6	32.3 (22.5;42.2)	50.6 (39.4;61.8)	41.0 (30.7;51.3)	19.7 (12.9;26.5)	31.4 (20.0;42.7)	22.1 (13.4;30.7)	27.9 (20.0;35.8)	19.1 (10.4;27.8)	40.4 (28.3;52.4)	.120	> 100
**Bloating**	30.4 (18.3;42.5)	23.7 (12.7;34.7)	43.6 (31.2;56.0)	32.0 (19.2;44.8)	15.2 (5.8;24.5)	30.2 (17.5;42.8)	23.3 (11.6;35.0)	28.6 (14.1;43.0)	21.7 (9.1;34.0)	35.1 (19.7;50.5)	.961	> 100
**Digestive Symptoms**	32.4 (21.7;43.0)	22.7 (12.7;32.8)	77.6 (65.6;89.6)	80.7 (69.2;92.1)	34.9 (22.3;47.4)	44.4 (30.3;58.6)	29.2 (16.8;41.5)	34.1 (18.9;49.3)	23.5 (9.5;37.6)	35.1 (21.0;49.2)	.962	> 100
**Taste**	15.7 (6.4;24.9)	19.2 (9.3;29.1)	26.7 (12.6;40.8)	32.0 (17.2;46.8)	27.3 (11.4;43.2)	28.6 (12.8;44.4)	25.0 (13.5;36.5)	34.9 (19.0;50.9)	11.8 (0.6;22.9)	31.6 (17.0;46.1)	.276	> 100
**Indigestion**	29.4 (16.8;42.0)	34.3 (21.2;47.5)	55.1 (40.2;70.1)	61.3 (47.4;75.3)	28.8 (16.4;41.2)	42.9 (27.8;57.9)	30.0 (17.6;42.5)	39.7 (25.7;53.7)	19.6 (9.8;29.4)	38.6 (23.4;53.8)	.162	> 100
**Flatulence**	33.3 (22.3;44.4)	29.3 (18.4;40.2)	44.9 (32.4;57.4)	36.0 (21.0;51.1)	36.4 (21.5;51.2)	18.3 (9.5;27.2)	40.0 (26.9;53.1)	36.5 (23.8;49.2)	39.2 (22.2;56.2)	40.4 (23.4;57.4)	.105	> 100
**Weight loss**	32.4 (18.7;46.0)	33.3 (20.3;46.4)	32.0 (17.2;46.8)	28.0 (12.6;43.4)	31.8 (16.6;47.0)	50.0 (30.2;69.8)	35.0 (17.6;52.4)	34.9 (17.2;52.7)	19.6 (7.0;32.2)	40.4 (21.9;58.8)	.718	> 100
**Weakness in Arms and Legs**	21.6 (12.5;30.7)	22.2 (11.7;32.7)	54.7 (40.6;68.7)	60.0 (45.4;74.6)	39.4 (24.7;54.1)	54.0 (38.0;70.0)	33.3 (19.1;47.6)	55.6 (40.4;70.7)	19.6 (4.7;34.5)	52.6 (33.7;71.5)	.051	> 100
**Dry Mouth**	29.4 (19.2 (39.7)	38.4 (28.1;48.7)	68.0 (56.2;79.7)	66.7 (53.6;79.7)	34.9 (20.3;49.4)	42.9 (27.2;58.6)	31.7 (15.6;47.7)	50.8 (34.8;66.8)	29.4 (12.7;46.1)	38.9 (24.7;53.1)	.618	> 100
**Hepatic Symptoms**	28.4 (18.2;38.7)	30.8 (20.6;41.1)	21.2 (11.5;30.8)	19.3 (7.5;31.2)	10.3 (4.2;16.5)	8.3 (-0.1;16.7)	10.8 (1.6;20.1)	7.9 (1.0;14.9)	13.7 (4.3;23.1)	14.0 (4.9;23.1)	.801	> 100
**Altered Bowel Habits**	29.3 (18.3;40.3)	30.2 (19.2;41.2)	15.3 (5.7;24.9)	23.3 (9.2;37.5)	22.0 (10.1;22.8)	31.0 (17.6;44.3)	35.0 (20.4;49.6)	38.9 (25.5;52.3)	28.4 (14.8;42.1)	28.7 (13.6;43.8)	.507	> 100
**Body Image**	22.0 (12.9;31.2)	21.9 (13.0;30.7)	40.0 (28.2;51.8)	29.0 (18.5;39.5)	25.4 (13.1;37.7)	31.0 (17.6;44.3)	29.8 (15.8;43.9)	36.7 (21.9;51.4)	25.5 (14.6;36.4)	35.1 (22.1;48.0)	.475	> 100
**Trouble with Side Effects**	28.0 (17.0;38.9)	29.2 (19.1;39.2)	62.8 (53.7;71.9)	61.1 (48.3;74.0)	45.5 (32.2;58.7)	49.2 (32.6;65.8)	43.9 (29.7;58.0)	54.0 (36.8;71.1)	35.3 (22.2;48.4)	50.0 (33.9;66.1)	.488	> 100
**Future Worries**	78.1 (67.7;88.5)	65.7 (52.2;79.1)	73.1 (61.0;85.1)	66.7 (52.6;80.8)	63.6 (50.1;77.2)	69.8 (53.0;86.7)	55.0 (39.8;70.2)	65.1 (49.8:80.4)	54.9 (39.1;70.7)	73.7 (57.4;89.9)	.733	> 100
**Planning Activities**	40.6 (26.9;54.3)	44.4 (31.2;57.7)	62.8 (46.5;79.2)	68.1 (51.6;84.5)	40.9 (27.4;54.5)	46.0 (28.9;63.2)	28.3 (12.4;44.2)	50.8 (34.2;67.4)	29.4 (14.7;44.1)	49.1 (31.6;66.7)	.180	> 100
**Satistfaction with Health Care**	11.9 (4.0;19.8)	26.6 (14.4;38.7)	12.2 (4.6;19.8)	13.9 (3.5;24.2)	20.0 (6.5;33.5)	25.0 (12.3;37.7)	33.3 (17.1;49.5)	16.7 (4.2;29.2)	***27.8 (12.9;42.6)***	***5.9 (1.1;10.7)***	***.048***	> 100
**Sexuality**	27.5 (16.1;38.9)	14.5 (4.4;24.6)	17.8 (4.1;31.5)	22.2 (5.5;39.0)	24.5 (11.9;37.1)	11.5 (1.5;21.6)	30.2 (18.2;42.2)	7.7 (-1.1;16.5)	25.6 (11.0;40.1)	6.1 (-0.6;12.7)	.474	> 100

In the C30 questionnaire in symptom subscales like appetite loss, high values indicate high symptom burdens. Thus, appetite loss is higher in cohort A 1 week postoperatively. In addition, it should be mentioned that for all post-hospitalization visits the subscales fatigue, insomnia and diarrhoea showed clinically relevant differences (i.e., > 10 points) between cohorts, with cohort B always showing higher symptomatic burden. One week postoperatively the pain subscale showed markedly higher (meaning worse) values for cohort A.

In the functioning subscales, higher values indicate better function. Therefore, for the subscales with differences between cohorts, cohort A always shows better functioning postoperatively. In addition, for all post-hospitalization visits all functioning scales show clinically relevant higher QoL for cohort A, except in the social subscale 3 months postoperatively. After a decrease 1 week postoperatively, (due to surgery), all subscales reach preoperative level or even better for cohort A, whereas scores in cohort B do not reach preoperative levels, with the exception of emotional functioning and cognitive function to some extent.

### Quality of Life assessment according to EORTC QLQ-PAN26

Apart from significantly lower satisfaction with the health care in cohort A after 12 months (*p* = 0.007; after Repeated Measures ANOVA: F (1,19) = 4.445; *p* = 0.048; η^2^_p_ = 0.190), no other subscales of the PAN26 showed a significant difference between cohorts. However, it should be mentioned that most subscales show markedly higher symptomatic burden for patients in cohort B in the post-hospitalization phase. Exceptions are subscales for flatulence (with no differences between cohorts), and sexuality (with worse scores for cohort A). With regards to the time course of the subscales, again for both cohorts, patients showed worse values 1 week postoperatively. However, cohort A reaches at least preoperative levels after 3 months, and mostly shows even better levels after 12 months, with the exception of the dry mouth subscale, which does not change markedly over time. In cohort B, only pancreatic pain, indigestion, dry mouth, hepatic symptoms, altered bowl habits, satisfaction with health care and sexuality reach preoperative levels or better (Table 2).

### Calculation of number of cases needed for future studies

The number of cases needed strongly depends on the questionnaire and/or the respective subscales one is interested in (see also last column in Table 2). According to the data gathered here, at least 120 patients overall would be needed to demonstrate a significant difference between cohorts due to physiotherapy using the SPPB. Calculations for subscales physical functioning, role functioning and appetite loss of the C30 questionnaire revealed that our patient collective was large enough or at least near to it, with 19, 36, and 34 patients needed in each group, respectively. For most other subscales of the C30 questionnaire, around 100 patients overall would have been needed to establish statistical significance. Exceptions are the subscales nausea and vomiting (> 600 patients), dyspnoea (> 1400 patients), insomnia, and financial difficulties (> 750 each). All subscales of the PAN26 questionnaire need between 100 and 200 patients to establish statistical significance.

## Discussion

In this first randomized controlled trial investigating the effects of prescribed intensive physiotherapy or exercise with a follow-up of 12 months in pancreatic cancer patients no significant difference in most QoL questionnaires’ subscales was found. However, patients with prescribed intensive physiotherapy or exercise almost regain their physical condition after 3–6 months to baseline levels, despite chemotherapy being administered. This clearly recognizable trend was not statistically significant. However, this was not to be expected in this small, monocentric cohort. Patients under prescribed intensive physiotherapy or exercise showed significant differences especially in physical functioning and role functioning subscales. Our study further revealed that prescribed intensified physiotherapy or exercise is safe, feasible and has a high adherence in pancreatic cancer patients, even in the post-hospitalization phase.

The state-of the art treatment of resectable pancreatic cancer including surgery and chemotherapy reduces health related QoL of pancreatic cancer survivors [18, 29–32]. Patient’s QoL is severely restricted after resection for PDAC, since these patients have significantly lower QoL scores in the physical and psychological domains compared to healthy controls [33]. Intensified physical training has been shown to measurably increase health-related QoL in patients with cancer [7, 12, 14, 34]. Meta-analyses, mainly based on breast, colon and prostate cancer, have reported benefits of exercise during and following cancer treatment [13, 35–38]. Nevertheless, little evidence exists on long-term effects of training during and after pancreatic cancer treatment in regard with chemotherapy. Previous published studies including pancreatic cancer patients have shown that intensive or supervised physiotherapy, even after a period of 3 months, improve QoL in a few aspects, such as physical functioning, fatigue, and mental health [6, 7, 17, 31]. Furthermore, systematic reviews have demonstrated that supervised exercise promotes significant improvements in clinical, functional, and in some populations even survival outcomes, regardless of the type of cancer involved [36, 39].

Since the long-term effects and feasibility of intensive physiotherapy or exercise in pancreatic cancer patients are not studied yet, we can hereby show that motivated or prescribed physical activity is positively associated with improvements in physical function and QoL. Even if pancreatic cancer is a deadly disease, pancreatic cancer patients undergoing treatment should be motivated to active exercise training, preferably supervised training or prescribed exercises. In contrast to Steindorf et al. [17], we can report long-term benefits on physical functioning and health-related QoL even after 6 months follow-up period with prescribed home-based exercises. Our data demonstrate the need for exercise prescriptions to preserve or improve physical functioning during and after pancreatic cancer treatment, and we can show that it’s safe and feasible, given the fact that quite a number of the patients with pancreatic cancer present themselves with frailty or fatigue, sarcopenia and / or weight loss, which generally reduces patients’ QoL and thus having a negative effect on overall survival [9, 40]. Furthermore, no negative scores in patients undergoing chemotherapy compared to those without chemotherapy was observed. Indeed, various QoL questionnaires’ subscales (i.e. physical functioning, role functioning and appetite loss) improved during follow-up in cohort A patients, even though 64% of them underwent adjuvant chemotherapy. Similar findings were reported by Cormie et al. and McLaughlin et al. [16, 41].

Of course, it might be debatable that after a follow-up of more than 6 months, the adherence to prescribed exercise might have reduced in both groups. However, adherence rate for those patients in the intervention group completing the whole follow-up, was around 80%. For example, the detection of local or distant recurrence in 48% of the study population may be one of the possible explanations in decline of motivation or adherence to exercise. However, exercise diaries provided by those patients who survived at least until the end of the follow-up time indicate that motivation stayed constant throughout follow-up. For the control group we know that at least 60% of all patients completing the follow-up took up the exercise regimen they were used to prior to surgery. In addition, one might argue that patient characteristics like age, ASA score or UICC status might influence the results presented here. Nonetheless, there were no obvious differences between groups with regards to those parameters, with the exception of reoperations needed (cohort A: 2; cohort B: 7). Though, this might have influenced QoL scores 1 week postoperatively, but should have no influence on the scores after 3 months. The drop-out of up to 50% during the follow-up period of 12 months was mostly due to death of the patients. However, our data also reflects the difficulty of implementing a supervised and/or prescribed intensive physiotherapy or exercise in the daily routine in patients with pancreatic cancer due to short overall survival. It is imaginable that there may be a patient self-selection bias regarding patients who finished the 12 months follow-up, as these patients were highly motivated and may have higher baseline fitness compared to the drop-out group, which may not represent all pancreatic cancer patients.

There are a few limitations in this study. It is a single-centre study with relatively few participants. However, number of cases calculations show, that at least with regards to the most important aspect physical functioning, the number of participants was large enough for a valid statement. The patients’ pre-diagnosis physical activity status may have influenced the initial motivation for participating in the study, and may thus present a selection bias we have not accounted for. Nevertheless, general activities during the course of follow-up are comparable across groups according to the diaries, even though no follow-up appointments between discharge and the 3-month follow-up were implemented. A monitoring using shorter intervals would have been interesting here in order to be able to observe improvements more closely. However, this was not possible due to the subsequent rehabilitation measures.

Future, randomized trials, preferably multi-centre, should aim to consistently assess the effects on long-term supervised exercise against home-base exercise, or even mobile app-based exercises, during and after pancreatic cancer treatment, not only on QoL per se but on clinical outcomes such as adherence and overall survival. In lieu of the drop-out rate in this study of up to 50% for a follow-up period of 12 months, and number of cases needed for most relevant subscales of the QoL questionnaires implemented here, we recommend a study population size of up to 400 patients.

The strengths of our study are noteworthy: 1. This is the first randomized controlled study with a 12-month follow-up in pancreatic cancer patients with a focus on QoL, 2. The effect of possible confounders such as adjuvant chemotherapy were accounted for as there were no notable differences between groups with regards to relevant patient characteristics, and 3. Doing a per protocol analysis, to only include those patients who were able to complete the whole follow-up time to avoid bias due to declining health and eventual death during follow-up.

In summary our study underlines two factors; first that prescribed home-based exercise and supervised exercise or training is safe and feasible after resection due to pancreatic cancer, and secondly, we are able to contribute to the fact that prescribed physiotherapy or exercise improves QoL in regard with some health-related aspects, especially during chemotherapy. Most remarkably, physical functioning improved significantly after 3–12 months in the intensive physiotherapy group. Therefore, our study provides additional evidence to support the implementation of exercise as part of standard care to improve health-related QoL. In addition, we could show that global QoL measures (SF-8 and global QoL of the C30) seem not specific enough to account for differences due to intensive physiotherapy, whereas certain aspects of health related QoL seem quite sensitive to physiotherapy after pancreatic resection. In contrast, most aspects measured by the PAN26 questionnaire seem a little bit too specific to the disease itself, as to be sensitive to the type of physiotherapy implemented.

## Conclusion

According to our data supervised exercise in pancreatic cancer patients and survivors may be proposed and started as soon as possible in order to improve certain aspects of QoL, since early controlled implementation seems to be safe. Also, benefits on some symptoms including physical and psychological aspects have been proved in this study. The impact on physical functioning is indeed so strong that even the small sample of this study was sufficient to demonstrate its significant effect. More data are needed to determine the optimal exercise frequency and intensity based on each patient´s training capacity or tumour stage, as a precision medicine approach is essential to address the issues of QoL. Well-designed multicentre randomized controlled trials with study populations between 20 and 1400 patients are required to examine the long-term effects of supervised and home-based exercise interventions on certain aspects of health related QoL after pancreatic cancer surgery.

## Data Availability

The datasets generated and/or analysed during the current study are not publicly available due to ethical and data security reasons (e.g., potential for identification of individual patients) but are available from the corresponding author on reasonable request.

## Abbreviations

ANOVA: Analysis of Variance
ASA: American Society of Anesthesiologists Physical Status classification
BMI: Body Mass Index
CI: Confidence Interval
CONSORT: Consolidated Statement of Reporting Trials
CRM: Circumferential resection margin
CTx: Chemotherapy
EORTC: European Organization for Research and Treatment of Cancer
ICU: Intensive Care Unit
IPMN: Intraductal Papillary Mucinous Neoplasm-Associated Carcinoma
NET: Neuroendocrine Tumour
QoL: Quality of Life
QLQ-C30: Quality of Life Questionnaire for Cancer patients developed by the EORTC
QLQ-PAN26: Additional pancreatic cancer specific module to the QLQ-C30
SD: Standard deviation
SF-8: Short Form-8 Health Survey
SPPB: Short Physical Performance Battery
UICC: Union for International Cancer Control
